# How Consumers in the UK and Spain Value the Coexistence of the Claims Low Fat, Local, Organic and Low Greenhouse Gas Emissions

**DOI:** 10.3390/nu12010120

**Published:** 2020-01-01

**Authors:** Faical Akaichi, Cesar Revoredo Giha, Klaus Glenk, Jose Maria Gil

**Affiliations:** 1 Department of Rural Economy, Environment and Society, Scotland’s Rural College, Edinburgh EH9 3JG, UK; cesar.revoredo@sruc.ac.uk (C.R.G.); Klaus.glenk@sruc.ac.uk (K.G.); 2 CREDA-UPC-IRTA, 08860 Barcelona, Spain; chema.gil@creda.es

**Keywords:** health, local, organic, greenhouse gas emissions, consumer, choice experiment, willingness to pay, trade-offs

## Abstract

This study investigates the substitution and complementary effects for beef mince attributes drawing on data from large choice experiments conducted in the UK and Spain. In both countries, consumers were found to be willing to pay a price premium for the individual use of the labels “Low Fat” (UK: €3.41, Spain: €1.94), “Moderate Fat” (UK: €2.23, Spain: €1.57), “Local” (UK: €1.54, Spain: €1.61), “National” (UK: €1.33, Spain: €1.37), “Organic” (UK: €1.02, Spain: €1.09) and “Low Greenhouse Gas Emissions (GHG)” (UK: €2.05, Spain: €0.96). The results showed that consumers in both countries do not treat desirable food attributes as unrelated. In particular, consumers in Spain are willing to pay a price premium for the use of the labels “Local”, “Organic” and “Low GHG” on beef mince that is also labelled as having low or moderate fat content. By contrast, consumers in the UK were found to discount the coexistence of the labels “Low Fat” and “Organic”, “Low Fat” and “Low GHG” and “Moderate Fat” and “Low GHG”. The results, however, suggest that in the UK the demand for beef mince with moderate (low) fat content can be increased if it is also labelled as “Organic” or “Low GHG” (“Local”).

## 1. Introduction

The prevalence of overweight and obesity is increasing at an alarming rate. It is estimated that approximately 2 billion adults are overweight and over 600 million are obese globally [1,2]. The increasing prevalence of overweight and obesity is placing a considerable burden on the economy and public health, including increases in the risk of developing serious health conditions, with direct healthcare costs amounting to billions [3,4,5].

Fortunately, obesity is preventable due to its strong, although not exclusive, link to diet. In fact, there is strong evidence that the prevalence of overweight and obesity is linked to the growing consumption of energy-dense foods and sugar-loaded beverages that are generally inexpensive, palatable and convenient [6,7,8]. As a result, it has been recognised that changing dietary habits and lifestyle would contribute to the reduction in overnutrition and its serious health and economic consequences [9,10,11,12].

In response, a whole raft of policy approaches have been designed and implemented to promote healthy diets and make the food selection environment more conducive to healthy choices. These approaches include mandates, restrictions, economic incentives, marketing limits, information provision and health campaigns [13,14]. Among these policy approaches, nutrition labelling is probably the most studied population-based health approach [15,16,17,18,19,20]. In general, these studies found that getting consumers to choose and eat healthier foods is not a trivial task. On the one hand, nutrition labels and health claims were found to have the potential to increase consumers’ demand for a healthier diet and help them to make more informed food choices. On the other hand, difficulty in understanding traditional nutrient declarations, especially those provided on the back of product packaging, was the most cited barrier to the use of nutrition labels.

Another aspect that may reduce the effectiveness of nutrition labels is the fact that this type of label is competing with other food attributes for consumer awareness. In fact, in addition to traditional food attributes such as price, income, taste and convenience, consumers are increasingly showing interest in less tangible food attributes, such as the sustainability, local origin, animal friendliness and social fairness of the production and processing of food products [21,22,23,24,25]. As a result, the strong interest in nutrition information exhibited by consumers in research studies may not translate into actual purchases of healthier food products. For example, a lamb consumer who is willing to pay a price premium for the labels “Local” and “Low Fat”, with the premium being higher for the former label, is likely to end up buying lamb labelled “Local” if the lamb carrying the label “Low Fat” is offered at the same or a higher price than the local lamb.

While extensive research has been devoted to assessing consumer understanding and use of nutrition labels and health claims, relatively little research has assessed how consumers weigh health-related food labels in comparison to labels for other desirable food attributes (such as organic, local, fair trade and high animal welfare) [21,24,25,26,27,28,29]. In general, it was found that despite the high interest shown by consumers in nutrition labels and health claims, it is possible that this interest does not translate into actual purchase, partly due to the trade-offs they make when choosing between food products with different desirable attributes.

Another factor that can affect (positively or negatively) the effectiveness of nutrition labels and health claims in increasing the demand for healthier foods is attribute bundling. In fact, because of consumers’ increasing interest in desirable attributes, such as organic, local and animal welfare, producers and marketers may bundle these attributes to increase their products’ differentiation, satisfy the needs of a larger number of ethically minded consumers and increase their sales. However, bundling desirable attributes is only a plausible strategy if consumers perceive them as independent or complementary. In other words, their value for the bundle of attributes is equal to or greater than the sum of their values for each individual attribute. If the desirable attributes are perceived as substituting or overlapping each other, bundling them will decrease consumers’ total marginal willingness to pay (WTP) for the bundle (A product is said to be complementary if it is used or consumed jointly with another product. Such a product usually has more value when paired with its complement than when used separately. A product is said to be a substitute for another product if it satisfies the same (or at least some of the) basic wants as the other product. Substitute products usually have more value when used separately than when used together.). For example, consumers can perceive the labels “Low Fat” and “Organic” as complementary if they think that the two labels refer to two complementary dimensions of food sustainability: health and environment. However, if consumers are expecting organic meat to have lower fat content, they may see the two labels as communicating partially overlapping information and, hence, discount the coexistence of both.

Most of the papers that investigated how consumers trade off different food attributes against each other assumed zero interactions between the attributes. Relaxing this assumption allows: (a) testing the effect of attribute bundling; and (b) correctly computing total consumers’ WTP for bundles of attributes. A food product is generally a bundle of different attributes. Consumers’ total WTP for the product is equal to the sum of their WTP for the individual attributes, forming the bundle, plus the value of the interactions between the bundled attributes. It is noteworthy that estimating the interactions between the attributes considered, for example, in a choice experiment, requires a larger number of observations and generally makes the estimation of a choice model with a high number (e.g., more than 10) of random main and interaction effects very challenging. Studies by Nilsson [27] and Bond et al. [28] were among the first to explicitly consider interaction effects between health-related attributes and other desirable attributes (e.g., organic, local).

Nilsson [27] found that the value of pork labelled as “Environmentally Certified” is enhanced if it also carries the label “Certified Free of Antibiotics”. However, they found that US pork consumers perceive the labels “Environmentally Certified” and “Certified for Animal Well-being”, and the labels “Certified for Animal Well-being” and “Certified Free of Antibiotics” as unrelated. Bond et al. [28] found that the coexistence of different health claims reduces the total marginal WTP for the bundles (i.e., perceived as substitutes). They found, however, that on top of price premiums for the labels “Organic” and “Excellent Source of Vitamin C”, consumers were willing to pay an additional premium for the coexistence of these two labels on the same product.

In addition to the policy and market implications of the findings of Nilsson [27] and Bond et al. [28], both studies provided evidence that focusing on the main effects of health-related attributes and ignoring their interactions with other desirable attributes may lead to biased and misleading results. This conclusion was also confirmed by other studies that looked at the interaction between non-health-related attributes [21,24,30].

This study contributes to the literature on how consumers trade off health-related attributes against other food attributes in three ways. First, we assess consumer preference and WTP for various labels of nonconventional attributes on beef mince products, with a focus on the labels “Low Fat”, “Moderate Fat”, “High Fat”, “Local”, “National”, “Imported”, “Organic”, “Low Greenhouse Gas Emissions (GHG)”, “Moderate GHG” and “High GHG”. To the best of our knowledge, this is the first study to investigate how consumers trade off the attribute fat content against the attributes origin, type of production and level of greenhouse gas emissions from production. Second, we investigate potential competition and complementarities between the labels “Low Fat”, “Moderate Fat”, “Local”, “Organic” and “Low GHG” to reveal much of the potential marketing information that could be used to promote healthier meat products. Third, we analyse how consumers’ preferences and WTP for individual and bundles of desirable food attributes vary by country (the UK versus Spain) and across consumer groups (gender and age group).

Therefore, this study aims to answer three empirical questions: (a) how do consumers perceive and value alternative health and “Sustainable” labels; (b) do they perceive these labels as unrelated or do they consider them as substitutes or complementary; (c) what degree of heterogeneity is there among consumers’ preferences and WTP?

## 2. Materials and Methods

The data were collected in the UK and Spain through a national web-based choice experiment. A choice experiment is a quantitative research technique that involves asking individuals to state their preference over hypothetical alternative scenarios, products or services. Each alternative is described by several attributes. Individuals’ responses are used to determine whether their preferences are significantly influenced by the attributes. The responses are also used to determine the relative importance of the attributes. Choice experiment has been used extensively in different research disciplines (e.g., marketing, health economics, environmental economics, the economics of transport) due to close resemblance to the real-world decision [31,32].

The initial design of the choice experiment was developed and revised based on input from a small sample of 110 respondents in each country. These respondents were not included in the dataset used for the econometric analysis. The final version of the survey was administered by a market research company. A total of 1211 and 1206 primary grocery shoppers in the UK and Spain, respectively, completed the survey. All subjects gave their informed consent for inclusion before they participated in the study. In both countries, the sample was required to be representative of the population in terms of gender, age, employment status and geographical area of the country. These hard quotas were achieved in both samples, except the age quota in the Spanish sample. In Spain, consumers aged 18 to 54 years old were slightly oversampled and consumers over the age of 54 were undersampled ((18−24) 11% vs. 8%; (25−34) 11% vs. 14%; (35−44) 28% vs. 19%; (45–54) 25% vs. 19%; (55+) 26% vs. 39%). Please note also that female consumers in both countries were slightly oversampled. This is because in EU, the majority of food buyers are female buyers). Because the product considered in this study is beef mince, only meat consumers were allowed to take part in the survey. The quality of the data was checked, and all the ineligible observations were discarded and replaced by eligible ones from new respondents. The socio-demographic characteristics of the two samples are provided in Table 1.

**Figure 1:**
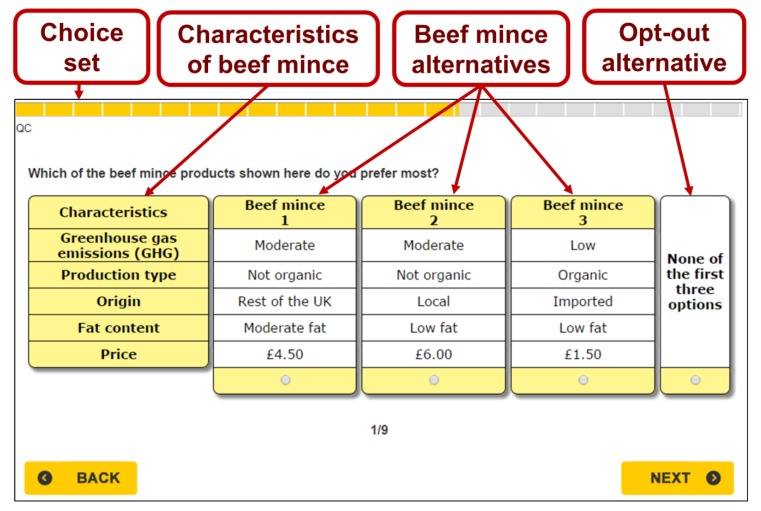
An example of a choice set used in the choice experiment conducted in the UK.

In each country, respondents were successively shown nine choice sets. Each choice set consists of three hypothetical beef mince alternatives and an opt-out alternative. An example of one of the choice sets used in the study is displayed in Figure 1. Each alternative of beef mince is described in terms of five attributes: fat content, type of production, origin, level of GHG emissions and price. The attributes and their corresponding levels were chosen based on the literature and the outcome of a shelf audit that was carried out in the major supermarkets in both countries. The attributes and their levels are described in Table 2.

Given all the attribute levels, a full factorial design of 216 (i.e., 3 × 2 × 3 × 3 × 4 = 216) profiles can be generated. Since presenting participants with 216 profiles would be time-consuming and cognitively very challenging, Ngene Software was used to generate a Bayesian D-optimal design that allows robust estimation of all main and two-way interaction effects [33]. The Bayesian D-optimal design was obtained after 25,000 iterations with 500 Halton draws per iteration, achieving a Db-error of 0.11 and 0.15 for the designs used in the UK and Spain, respectively. Each of the final designs of the choice experiment consisted of 36 choice sets of four alternatives each (i.e., three beef mince alternatives plus the opt-out alternative). To make the choice task cognitively easier for respondents, the design was blocked in four blocks (i.e., nine choice sets per respondent). In the choice task, respondents were successively shown nine different choice sets and were repeatedly asked to choose the alternative they prefer most.

**Figure 2:**
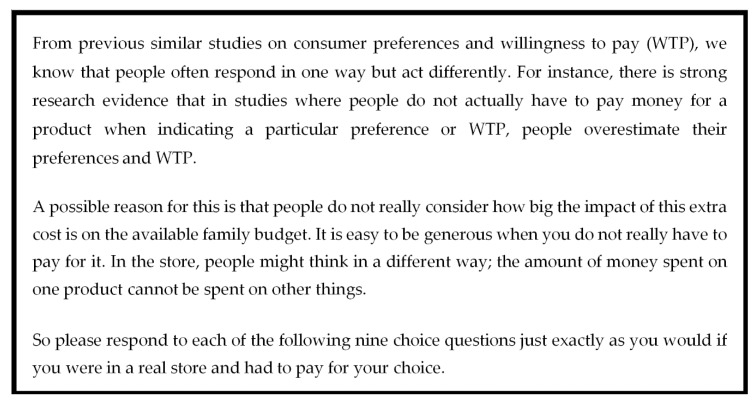
The cheap talk script used in this study.

**Figure 3:**
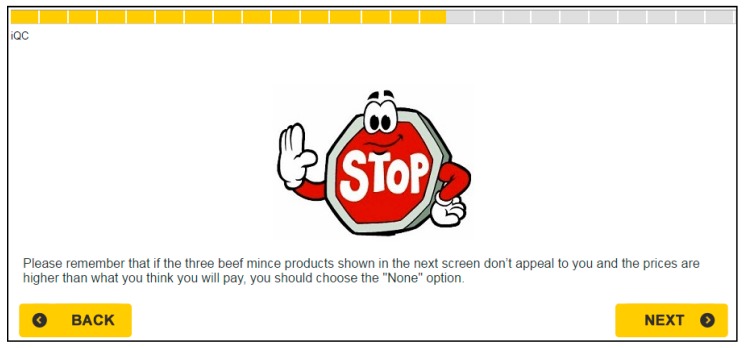
The opt-out reminder that was shown to respondents before each choice set.

To reduce the effect of hypothetical bias, we followed the approach used by Ladenburg, and Olsen [34]. This approach consists in including a cheap talk script [35] right before the choice task. Then the cheap talk script is augmented with a repeated opt-out reminder, showed to participants before each choice set. The cheap talk script and the opt-out reminder used in this study are displayed in Figure 2 and Figure 3, respectively.

Note that the order of showing the nine choice sets was randomised for each respondent. The choice task was followed by a questionnaire. The questionnaire was used to collect information on respondents’ socio-demographic characteristics as well as their purchasing habits and attitudes toward several food attributes (e.g., health, environmental sustainability, origin, price, labelling). The questionnaire was also used to collect information on various aspects of respondents’ choice behaviour such as attribute nonattendance, certainty about choice responses, and respondents’ level of altruism and free riding.

The data collected were analysed within a random utility framework [36]. Thus, an individual *n* presented with *j* alternatives at a choice occasion *t* is expected to choose the alternative that maximises his/her utility. Following Lancaster’s [37] concept that any product is a bundle of attributes, the utility that an individual *n* derives from the consumption of a product is assumed to be equal to the sum of his/her marginal utility for each of the product’s attributes. Consequently, if we assume a sample of *N* respondents who are presented with *T* choice occasions of *J* alternatives each, individual *n*’s utility ($U_{njt}$) from choosing the *j*th alternative at a *t*th choice occasion takes the form

(1) $$U_{njt}=V_{njt}+\varepsilon_{njt}$$

where $V_{njt}$ is the deterministic (observed) component and $\varepsilon_{njt\;}$ is the random (unobserved) component. $\varepsilon_{njt}$ is assumed to be independent and identically distributed. Assuming that the deterministic component of the utility is linear-in-parameter, Equation (1) can be written as

(2) $$U_{njt}=\beta X_{njt}+\varepsilon_{njt}$$

where β denotes the K × 1 vector of unknown utility parameters. As described in more detail further below in this study, $X_{njt}$ represent the following level of attributes “Low Fat”, “Moderate Fat”, “Local”, “Rest of the country”, “Organic”, “Low GHG”, “Moderate GHG” and “Price” as well as the six two-way interactions (“Low Fat and Organic”, “Low Fat and Local”, “Low Fat and Low GHG”, “Moderate Fat and Organic”, “Moderate Fat and Local”, “Moderate Fat and Low GHG”). (We were not able to estimate the choice model with all possible two-way interactions due to problems of convergence we had faced during the estimation of the choice model). The levels “High Fat”, “Imported”, “Not Organic (No label)” and “High GHG” were dropped from the estimation to avoid the problem of perfect multicolinearity. They are also used as the baseline levels when interpreting the estimated effects.

Conditional logit (CL) [36] is the workhorse model for analysing discrete choice data. However, its assumptions (i.e., homogeneity of respondents’ preferences and the independence of the alternatives included in any choice set) do not generally hold [32]. Revelt and Train [38] proposed a less restrictive model (Random Parameter Logit (RPL)) that allows individuals’ preferences to be heterogeneous and the assumption of the independence of alternatives to be relaxed. In the RPL, at least one parameter is specified as random. In other words, each individual is considered to have a unique set of preferences, reflected in the individual parameters $\beta_{i}$. Since the unconditional choice probability does not have a closed-form solution, simulation methods are used to estimate the parameters (see Revelt and Train [38] for details).

In this study, the parameters for all the non-price attributes as well as the six two-way interactions were assumed to be normally distributed. Theoretically, the estimated coefficient for the price is expected to be negative. Therefore, to avoid obtaining unrealistic positive values for the parameter price, we first multiplied the variable price by −1. Then, a lognormal distribution was imposed on the variable price instead of a normal distribution [39].

In addition to obtaining information on consumers’ preferences, the use of discrete choice models allows the derivation of measures designed to determine the amount of money individuals are willing to give up in order to obtain some benefit from the non-price attributes of the product (e.g., low fat, organic, local). Such measures are referred to as measures of WTP. The most used approach to calculate consumers’ WTP consists of computing the ratio of two estimated parameters, holding all else constant. In particular, WTP is commonly expressed as the negative ratio of the non-price attribute coefficient (e.g., the coefficient for the level organic) to the price coefficient:

(3) $$WTP_{non\text{-}price\;attribute}=-\frac{\beta_{non\text{-}price\;attribute}}{\beta_{price}}$$

The calculated value represents the respondents’ marginal WTP. In this study, the attributes’ levels considered in the estimation of the RPL model were all coded as dummies. Therefore, the calculated WTP value represents respondents’ marginal WTP for the attribute level considered in the estimation (e.g., “Low fat”) relative to the baseline level (e.g., “High fat”). Note that it is more robust to estimate consumers’ marginal WTP following the approach proposed by Train and Weeks [40]. Train and Weeks [40] proposed to estimate the RPL model in WTP space instead. This involves estimating the distribution of willingness to pay directly by re-formulating the model in such a way that the coefficients to be estimated represent the WTP measures. The estimation of the RPL model in WTP space is very time consuming for large sample size and high number of random parameters. We tried to estimate the RPL in WTP space but we had to abort the estimation after nine days running without reaching convergence.

In some cases, the estimated coefficient was found to have a statistically insignificant mean but significant standard deviation. A statistically significant standard deviation suggests that consumers’ preferences for the estimated effect are heterogeneous. This heterogeneity can be the cause of the nonsignificance of the estimated mean of the effect if consumers are equally split into both positive and negative sides of the preference scale. If this is the case, the positive and negative effects can cancel each other out, resulting in a statistically insignificant mean of the investigated effect. To test this hypothesis, we followed the approach mentioned in Train [41] to compute the percentage of respondents who placed a positive (or negative) value on the estimated effect using the following formula:

(4) $$100\times \Phi \left(\frac{-\beta_{k}}{S_{k}}\right)$$

where Φ is the cumulative standard normal distribution and *β_k_* and *S_k_* are the mean and the standard deviation of the *k*th interaction parameter, respectively. Note that this formula is only applicable if the random parameter of interest has a symmetric normal distribution.

After estimating the RPL model for the whole sample, we found that consumers’ preferences in both countries are strongly heterogeneous. To evidence the heterogeneity of consumers’ preferences and how they vary across different consumer groups, we estimated the RPL model for five consumer segments: “Women”, “Men”, “Youth (<30 years old)”, “Adults (30–60 years old)”, and “Elderly (>60 years old)”. So, in total, six RPL models were estimated. Furthermore, the standard error of consumers’ WTP was computed using the delta method [42].

To test whether the differences in respondents’ WTP in the different segments are statistically significant, the Complete Combinatorial Test, proposed by Poe et al. [43], was used. The test, first, requires the generation of distribution of 3000 WTP estimates using the parametric bootstrapping method proposed by Krinsky and Robb [44]. Then, the complete combinatorial test is used to compare the bootstrapped WTP values in the different segments.

## 3. Results

The RPL models were estimated using Stata 15, with 2000 Halton draws to simulate the 15 random parameters (i.e., nine main effects and six interaction effects). The results of the estimated marginal utilities (i.e., preferences) and their standard deviations, as well as the marginal WTP of the sampled respondents in the UK (Model 1) and Spain (Model 2), are presented in Table 3. The results show that the estimated RPL models for panel data fit the data better when tested against the basic conditional logit: χ^2^ = 2214.35, *p* < 0.01 in the case of the UK data and χ^2^ = 1718.10, *p* < 0.01 in the case of the data collected in Spain.

### 3.1. Main Effects

The results displayed in Table 3 show that the main effect coefficients are all significant at 1% and of the expected sign. Another general result worth mentioning is that the significance of the standard deviations of all the main effects (except for the label “Moderate Fat” in the UK model) shows that heterogeneity is indeed an issue to be considered when investigating consumers’ preferences for desirable attributes of beef mince. A binary variable “No Buy Option” was included in the estimation of the RPL models to capture respondents’ preferences for the opt-out alternative. In both models, the estimated coefficient is highly significant. The estimated coefficient was found to be positive in Model 1 and negative in Model 2. The coefficient is negative when more than two-thirds of respondents (77% in Spain versus 69.7% in the UK) have a higher preference for the beef mince alternative than for the opt-out alternative, and vice versa if the sign of the estimated coefficient is positive. The significant and negative value of the price coefficient in both models indicates that consumers in both countries prefer cheaper beef mince, all else being equal.

In both models, the positive coefficient value for the labels “Low Fat”, “Moderate Fat”, “Local”, “Rest of the Country”, “Organic”, “Low GHG” and “Moderate GHG” indicates that the utility associated with beef mince packages carrying these labels is higher than the utility for the labels set as baseline (i.e., “High Fat”, “Imported”, “Not Organic” and “High GHG”). In particular, the results suggest that consumers in the UK and Spain favour beef mince labelled as “Low Fat” or “Moderate Fat”, as opposed to beef mince carrying the label “High Fat”. Note that respondents in both countries prefer beef mince with low fat content over beef mince with moderate fat content.

Regarding respondents’ preferences for the labels communicating information about the origin of the beef mince, in both models consumer utility was found to be the highest for local beef mince, followed by national and imported beef mince in second and third place, respectively. The majority of sampled respondents in the UK and Spain were found to prefer organic beef mince as opposed to its conventional counterpart. Consumers in both countries were found to be more likely to choose beef mince that carries the labels “Low GHG” or “Moderate GHG” as opposed to beef mince that carries the label “High GHG”. While respondents in the UK have a higher preference for beef mince labelled as “Low GHG” than beef mince that carries the label “Moderate GHG”, respondents in Spain showed higher preferences for the label “Moderate GHG” than the label “Low GHG”.

Regarding respondents’ preferences for the labels communicating information about the origin of the beef mince, in both models consumer utility was found to be the highest for local beef mince, followed by national and imported beef mince in second and third place, respectively. The majority of sampled respondents in the UK and Spain were found to prefer organic beef mince as opposed to its conventional counterpart. Consumers in both countries were found to be more likely to choose beef mince that carries the labels “Low GHG” or “Moderate GHG” as opposed to beef mince that carries the label “High GHG”. While respondents in the UK have a higher preference for beef mince labelled as “Low GHG” than beef mince that carries the label “Moderate GHG”, respondents in Spain showed higher preference for the label “Moderate GHG” than the label “Low GHG”.

Despite their usefulness in investigating how consumers weigh different food attributes and in predicting the probability of possible future choices, estimated utility coefficients do not provide direct information for welfare and other policy analyses. Instead, they are commonly converted into money values (e.g., WTP). Respondents’ marginal WTP for the different labels considered in this study and their interactions are displayed in the last three columns of Table 3. The results show that the labels “Low Fat” and “Moderate Fat” are highly valued by consumers in both countries. Nonetheless, UK consumers’ price premiums for the labels “Low Fat” and “Moderate Fat” are respectively 76% (€3.41 versus €1.94) and 42% (€2.23 versus €1.57) higher than the price premiums of Spanish consumers. Regarding the value consumers give the origin-related labels, the sampled consumers in both countries have statistically similar marginal WTPs for the beef mince labelled as “Local” or “Rest of the UK” / “Rest of Spain”. However, note that respondents in the UK and Spain are willing to pay 16% (€1.54 versus €1.33) and 18% (€1.61 versus €1.37) more, respectively, for the label “Local” than for the label “Rest of the UK” / “Rest of Spain”.

Consumers in the UK and Spain were found to be willing to pay a comparable price premium for beef mince labelled “Organic”. In both countries, consumers’ price premium for the label “Organic” is significantly lower than their price premiums for five of the labels considered in the analysis. For example, in the UK, consumers’ price premium for organic beef mince is 234%, 119%, 101%, 51% and 30% less than their price premiums for the labels “Low Fat”, “Moderate Fat”, “Low GHG”, “Local” and “Rest of the UK”, respectively. In both countries, beef mince carrying the labels “Low GHG” or “Moderate GHG” received a significantly higher value than beef mince labelled as “High GHG”. Nonetheless, in the UK, consumers value the label “Low GHG” more (+133%) than the label “Moderate GHG”, as opposed to consumers in Spain, who value beef mince labelled as “Moderate GHG” more highly (29%) than beef mince that carries the label “Low GHG”. Note that, when compared with the label “Organic”, consumers in the UK value the label “Low GHG” more highly, while consumers in Spain value “Moderate GHG” more highly. Furthermore, the value UK consumers give the label “Low GHG” is 114% higher than the value consumers in Spain give it. However, UK consumers’ price premium for beef mince labelled as “Moderate GHG” is 41% lower than Spanish consumers’ price premium for the same label.

### 3.2. Interaction Effects

In line with the findings of previous studies [21,24,25,28,29,30], the results from the estimation of the main effects show that consumers in the UK and Spain value the labels “Low Fat” and “Moderate Fat” highly and they are also willing to pay an economically significant premium for the labels “Local”, “Organic” and “Low GHG”. As explained in the introduction, a key contribution of this study to the literature on consumers’ preferences and WTP for healthier food products is its assessment of whether bundling health-related labels with other positively valued labels (e.g., “Local”, “Organic” and “Low GHG”) can boost the demand for healthier beef mince. To answer this question, six two-way interactions were estimated (“Low Fat and Organic”, “Low Fat and Local”, “Low Fat and Low GHG”, “Moderate Fat and Organic”, “Moderate Fat and Local” and “Moderate Fat and Low GHG”). The estimated marginal utilities and WTP for the six interactions are presented in Table 3.

Contrary to the results of the estimated main effects, the estimated interaction parameters do not have the same sign across countries. In the case of Spain, all the estimated interactions are positive and statistically significant. This indicates that consumers in Spain perceive the labels constituting the six bundles as complementary. This, in turn, implies that they are willing to pay a significant price premium for the coexistence of the bundled labels in addition to the price premium they are willing to pay for the individual labels. For example, if the beef mince that carries the label “Low Fat” is also labelled as “Local”, consumers’ total willingness to pay for the bundle is equal to their price premium for labels “Low Fat” (€1.94) and “Local” (€1.61) plus their price premium for the coexistence of the two labels on the same product (€0.24). Therefore, it can be deduced that Spanish consumers’ total marginal WTP for beef mince that carries the bundle of labels “Low Fat and Local” equals €3.79, “Low Fat and Organic” equals €3.32, “Low Fat and Low GHG” equals €3.71, “Moderate Fat and Local” equals €3.62, “Moderate Fat and Organic” equals €2.88 and “Moderate Fat and Low GHG” equals €3.10. It is noteworthy that with the exception of the interaction “Low Fat and Local”, the estimated standard deviations for the other five interactions are statistically insignificant. This implies that consumers’ positive valuation of the coexistence of the labels constituting the bundles “Low Fat and Local”, “Low Fat and Organic”, “Low Fat and Low GHG”, “Moderate Fat and Local”, “Moderate Fat and Organic” and “Moderate Fat and Low GHG” are highly homogeneous. Overall the results suggest that, in the case of Spain, adding the labels “Local”, “Organic” or “Low GHG” to beef mince that is labelled “Low Fat” or “Moderate Fat” can boost the demand for it.

In the case of the UK, the results show that only the coexistence of the labels “Low Fat” and “Local” is valued positively (i.e., the two labels are perceived as complementary). In particular, UK consumers are willing to pay an additional premium of €0.25 for beef mince that is labelled as “Low Fat” and “Local” at the same time. UK consumers were also found to perceive the labels constituting the bundles “Low Fat and Organic”, “Low Fat and Low GHG” and “Moderate Fat and Low GHG” as (partial) substitutes. In particular, consumers discount their total marginal WTP for beef mince that is simultaneously labelled as “Low Fat” and “Organic” by €0.38. This is equivalent to offsetting 37% of consumers’ price premium for the label “Organic” (i.e., €0.38/€1.02) *100). Similarly, labelling beef mince that carries the label “Low Fat” or the label “Moderate Fat” as “Low GHG” is expected to reduce consumers’ total price premium for the bundles “Low Fat and Low GHG” and “Moderate Fat and Low GHG” by €1.09 and €0.98, respectively. This is equivalent to offsetting the positive impact of the label “Low GHG” by 53% when bundled with the label “Low Fat” and by 48% when bundled with the label “Moderate Fat”.

The estimated coefficients of the interactions “Moderate Fat and Local” and “Moderate Fat and Organic” are statistically insignificant. This result seems to suggest that UK consumers perceive labels constituting the bundles “Moderate Fat and Local” and “Moderate Fat and Organic” as unrelated. Nonetheless, the estimated standard deviations of the two interactions are statistically significant, suggesting that consumers’ preferences for these bundles are heterogeneous. As noted above, this heterogeneity can be the cause of the nonsignificance of the estimated interaction parameters if consumers are equally split into both positive and negative sides of the preference scale. To compute the percentage of respondents who gave a positive (or negative) value to the bundles “Moderate Fat and Local” and “Moderate Fat and Organic”, we used the formula in Equation (4).

We found that, in the case of the bundle “Moderate Fat and Local”, 56% of UK consumers perceived the labels “Moderate Fat” and “Local” as substitutes, while 44% of them perceived them as complementary. In the case of the bundle “Moderate Fat and Organic”, we found that while 59% of UK consumers positively valued the coexistence of the labels “Moderate Fat” and “Organic”, 41% of them valued it negatively. These results suggest that the heterogeneity of consumers’ preferences is possibly behind the nonsignificance of the estimated interaction parameters for the bundles “Moderate Fat and Local” and “Moderate Fat and Organic”. However, we recommend using these results with caution due to the strong assumptions and low robustness of the approach proposed by Train [41] used here.

### 3.3. Heterogeneity of Consumers’ WTP

As noted above, the significance of the estimated standard deviations (Table 3) indicates that consumers’ preferences and marginal WTP are heterogeneous, especially for the main effects. The results from the estimation of the RPL models for the segments “Women”, “Men, “Youth (<30 years old)”, “Adult (30–60 years old)” and “Elderly (>60 years old)” are displayed in Table 4 (for the UK) and Table 5 (for Spain).

The results show that gender and age can partially explain the heterogeneity of consumers’ preferences and WTP. The results in Table 4 show that, in the UK, female consumers are willing to pay a significantly higher price premium for the labels “Low Fat”, “Local” and “Moderate GHG”. Both female and male consumers valued the label bundles considered in this study similarly, with the exception of the bundle “Moderate Fat and Organic”. While male respondents perceived the labels “Moderate Fat” and “Organic” as independent, female consumers considered the two labels to be complementary and were willing to pay an additional premium of €0.40 for beef mince that is simultaneously labelled as “Moderate Fat” and “Organic”. Interestingly, in the case of Spain, the results in Table 5 show that female and male consumers have similar preferences and WTP for the main effects (except for the label “Low Fat”). The results for the interaction effects show that while female consumers are willing to pay an additional premium for all the bundles (except “Low Fat and Local”), male respondents in Spain seem to perceive the labels constituting the bundles “Low Fat and Low GHG”, “Moderate Fat and Organic” and “Moderate Fat and Low GHG” as independent.

With regard to the differences across age segments, the results in Table 4 show that UK respondents over the age of 60 are willing to pay the highest premium for the labels “Low Fat”, “Moderate Fat”, “Local”, “Rest of the Country”, “Low GHG” and “Moderate GHG”. Young UK consumers’ price premium for the beef mince labelled as “Organic” is the highest among the three age segments. For the interaction effects, the results from the data collected in the UK show that consumers’ preferences and WTP are similar among the three age segments, except for the bundles “Low Fat and Local” and “Low Fat and Low GHG”. While older respondents perceived the labels “Low Fat” and “Local” as complementary, young and adult consumers perceived them as unrelated. Furthermore, while young and elderly respondents considered the labels “Low Fat” and “Organic” to be independent, adult consumers would discount the total marginal WTP for beef mince simultaneously labelled as “Low Fat” and “Organic” by €0.52.

As opposed to UK consumers, young consumers in Spain (Table 5) were found to have the highest price premium for the labels “Low Fat”, “Moderate Fat”, “Local”, “National”, “Low GHG” and “Moderate GHG”. While elderly UK consumers were willing to pay the lowest price premium for organic beef mince, elderly respondents sampled in Spain were found to be willing to pay the highest price premium for beef mince labelled as “Organic”. The results in Table 5 show that adult consumers in Spain perceive the labels forming the bundles “Low Fat and Organic”, “Low Fat and Local”, “Low Fat and Low GHG”, “Moderate Fat and Organic” and “Moderate Fat and Local” as complementary, as opposed to the young and elderly consumers who consider these labels to be unrelated.

## 4. Discussion and Conclusions

The results suggest that labels indicating that the beef mince has a low or moderate fat content are of significant importance for consumers in the UK and Spain. This finding is in line with the dietary recommendations of the UK and Spanish governments [45,46,47], as well as the World Health Organisation [48], aiming to curb the epidemic prevalence of obesity through encouraging consumers to eat fewer food products that are high in fat, sugar or salt. Previous studies [25,49,50] similarly found that consumers in different European countries prefer their food to have a low fat content.

Despite the high interest in beef mince carrying the label “Low Fat”, producing and selling low-fat beef mince seems to be challenging. In fact, the authors carried out an online shelf audit of the major supermarkets in the UK (Asda, Tesco, Sainsbury’s and Waitrose) and Spain (Carrefour, Alcampo, Eroski Caprabo, Mercadona and Corte Inglés) to check the availability and the retail prices of beef mince with a low or moderate fat content. We found that in both countries, none of the beef mince products sold online can be technically labelled as low in fat (i.e., containing 3 g or less of fat per 100 g of beef mince). Nonetheless, in both countries, most of the beef mince products sold online have a moderate level of fat. We also noticed that in Spain, fat-content-related labels are only occasionally displayed on the front of packages of beef mince, as opposed to the UK where the use of front-of-package health labels and claims is more common. The reasons (e.g., taste, cost, technical and processing factors) for this very limited availability of beef mince with a fat content of less than 3% in grocery stores, as well as the uncommon use of front-of-package nutrition labels in Spain still need to be studied in future research. Uncovering these reasons should help to improve the supply and marketing of low-fat beef mince, thus satisfying the needs of an increasing number of consumers interested in health.

We also learned from the online shelf audit that beef mince with a moderate fat content is sold at a retail premium that ranges between €3.40 and €6.20 per kg in the UK and it does not exceed €3 per kilogramme in Spain. These retail premiums are close to the estimated price premiums that the sampled consumers in the UK (€4.46) and Spain (€3.14) are willing to pay for beef mince labelled as “Moderate Fat”. However, a more rigorous analysis that makes use of data with a good temporal and spatial coverage of beef mince’s retail prices is needed to find out whether healthier beef mince products are currently sold at affordable prices. Our results clearly show that consumers in the UK are willing to pay a significantly higher price premium for healthier beef mince than consumers interviewed in Spain. This suggests that marketers who are interested in selling beef mince in both countries should consider the substantial gap between consumers’ price premiums for healthier beef mince in the two countries (note that consumers’ WTP in both countries was not adjusted for the differences between the two countries in terms of food prices, consumers’ income, etc.).

In line with the results of several previous studies [21,22,23,24,25], we found that the majority of sampled consumers in the UK and Spain also positively value the labels “Local”, “Rest of the Country”, “Organic”, “Low GHG” and “Moderate GHG”. Interestingly, in both countries, consumers’ price premium for beef labelled as “Organic” is significantly lower than their price premium for beef mince that carriers the label “Local” or “Rest of the Country”. Onozaka and McFadden [21] and Meas et al. [24] similarly found that US consumers have a higher preference for local food than organic food. While our paper does not investigate the reasons for the higher preferences for local beef mince (see [51,52]), it suggests that labelling locally (nationally) produced and sold beef mince as “Local” (“National”) can increase its competitive power vis-à-vis organic and imported beef mince.

The results also suggest that there is a potential market in the UK and Spain for beef mince produced with low or moderate greenhouse gas emissions. Carbon labels are not currently used in the UK and Spain to label meat and meat products, especially those produced with low GHG emissions. This is probably due to the high carbon footprint of most of the meat produced and sold in both countries, as well as the difficulty of continuously collating precise data on GHG emissions from meat production [53,54]. As a result of the increasing calls to reduce meat consumption [55], meat producers and processors are under unprecedented pressure to reduce the environmental impact of their products. In this context, our results are sending a positive signal back to producers and marketers of beef mince that consumers are willing to bear a considerable increase in beef mince price (up to €2.05 in the UK and €1.24 in Spain) if the product is produced with a significantly lower environmental impact.

Despite consumers’ high price premium for beef mince labelled as “Low Fat” or “Moderate Fat”, the results showed that consumers in both countries may trade off this beef mince against a counterpart that is carrying other desirable labels, such as “Local”, “Organic” and “Low GHG”. This competition is further increased if the non-health-related labels are bundled. For example, the results suggest that if beef mince products labelled “Moderate Fat”, “Local” and “Organic” are offered at the same price, consumers in the UK and Spain are more likely to buy the beef mince with a moderate fat content because of their higher price premium for this type of beef mince. Nonetheless, if the organic beef mince is also labelled as “Local” when sold in the local market, consumers in both countries may purchase the locally produced organic beef mince instead (UK: €2.56 versus €2.23, Spain: €2.70 versus €1.57). To sum up, when pricing and marketing their products, producers and marketers of healthier beef mince should take into account the trade-offs that consumers may make when they are faced with products carrying other desirable food labels such as “Local” and “Organic”.

Bundling desirable attributes can also boost the demand for healthy food products if consumers perceive the attributes bundled to be complementary or independent. In this study, consumers were found to positively value the labels “Low Fat”, “Moderate Fat”, “Local”, “Organic” and “Low GHG”. This implies that bundling these labels can improve the demand for beef mince if consumers perceive them as complementary or independent. Nonetheless, if they are perceived as substitutes, it implies that consumers negatively value the coexistence of the bundled labels. The results show that sampled consumers in Spain think that the labels “Local”, “Organic” and “Low GHG” complement the labels “Low Fat” and “Moderate Fat”. This suggests that the demand for low- or moderate-fat beef mince can be improved if it is also labelled as “Local”, “Organic” or “Low GHG”. Therefore, it is recommended that Spanish producers and marketers of beef mince with low or moderate fat content also label their product as local when it is sold in local markets. The findings of this study suggest that producers of organic beef mince can also benefit from reducing the fat content of their product and promote this fat reduction by labelling the organic product as “Low Fat” or “Moderate Fat”.

The results from the data collected in Spain show that the coexistence of the labels “Low Fat” and “Low GHG” received the highest price premium. Therefore, the demand for low-fat beef mince in Spain can be increased if the product is also labelled as produced with low GHG emissions. However, it remains to be seen whether producing low-fat beef mince, which may necessitate the use of a higher quantity of beef meat, is feasible with a low environmental impact. The coexistence of the labels “Moderate Fat” and “Low GHG” was also positively valued by the sampled consumers in Spain. This is further encouraging evidence of the existence of potential demand for beef mince with a low carbon footprint. Most importantly, this also suggests that the producers and marketers of beef mince with moderate fat content, which is currently the most marketed type of beef mince in Spain, can receive higher price premiums if they manage to significantly reduce the total environmental impact of their product.

Sampled consumers in the UK were found to value the coexistence of multiple labels differently in comparison with consumers in Spain. The only exception is the coexistence of the labels “Low Fat” and “Local”. UK consumers were found to positively value the coexistence of the two labels on the same beef mince product, suggesting that UK producers and marketers of low-fat beef mince can increase their sales in the local market if they also label their product as “Local”. In contrast with the preferences of the sampled consumers in Spain, UK consumers were found to discount the simultaneous use of the label “Low GHG” or the label “Organic” with the labels “Low Fat” and “Moderate Fat”. Whilst producers and marketers of low- or moderate-fat beef mince should still label their products “Organic” or “Low GHG”, it is recommended that they take into account the value of the discount when computing the total return of the discounted bundles (e.g., “Low Fat and Organic”).

Furthermore, consumers in the UK were found to perceive the labels constituting the bundles “Moderate Fat and Local” and “Moderate Fat and Organic” as unrelated. This implies that the total price premium for the bundle is equal to the sum of the price premiums for the individual labels. This is noteworthy for at least two reasons. First, producers and marketers of beef mince with moderate fat content can increase the demand for their product if they label it as “Local” when it is sold in local markets. The results also suggest that the demand for organic beef mince can be boosted if it is labelled as having a moderate fat content. Second, moderately reducing the fat content of beef mince (to be between 3.1% and 20% of fat) is technically and financially more feasible than producing beef mince with less than 3% fat content. In fact, most of the beef mince that is currently sold in the UK and Spain is eligible for the label “Moderate Fat” without any further processing.

As mentioned in the introduction, most of the research studies that have investigated individuals’ preferences and willingness to pay for food products with multiple attributes assumed that respondents treat food attributes as unrelated. Our findings clearly show that this assumption is inappropriate and can lead to biased and misleading results, which in turn is likely to bias the output from subsequent analyses such as cost–benefit analysis. This is on top of ignoring important information on how consumers trade off food products with multiple attributes. In this study, we found that only two out of 12 estimated interaction effects were insignificant. Therefore, we join Onozaka and McFadden [22] and Meas et al. [24], among others, in encouraging researchers who want to investigate individuals’ preferences and choices in multiple attribute settings to design the choice experiment in a way that they can later at least estimate the two-way interaction effects.

Previous studies (e.g., [23,24,25]) investigated the heterogeneity of consumers’ preferences for food products with desirable attributes. They found that consumers’ preferences and WTP for food products labelled as healthier, local or organic vary by age, income level, education level and gender. In line with those studies, we found that consumers’ preferences are heterogeneous across countries and consumers within the same country. For example, we found that while elderly people in the UK constitute the group of consumers with the highest price premium for the desirable attribute levels, young consumers in Spain seem to be the segment with the highest demand for beef mince carrying desirable labels such as “Low Fat”, “Moderate Fat” and “Local”. The results also highlight the importance of assessing the heterogeneity of consumers’ preferences, especially if the outcome of the assessment is intended to help in designing effective policy and marketing strategies that are tailored to the needs of different consumer groups.

Like any other empirical study, the research work described in this paper has some limitations. For example, the data was collected using web-based survey. The use of web-based surveys has many advantages such as ease of data gathering, minimal costs, automation in data input and handling, increase in response rates, flexibility of design, potentially better targeting and convenience to participants. However, one should be aware of the drawbacks of online surveys. Perhaps the heaviest disadvantage of online surveys is the difficulty to avoid getting answers from respondents who answer online surveys mainly for getting the monetary incentive and not with a desire to contribute to the advancement of the study by reporting honest answers. As noted in the methods’ section, we used some incentives (e.g., cheap talk, opt-out reminder and required minimum time for evaluating each choice card) to reduce the occurrence of this problem.

These incentives were also used to reduce the effect of hypothetical bias, which is another typical limitation of the use of web-based surveys. Hypothetical bias occurs because respondents tend to respond differently to hypothetical scenarios than they do to the same scenarios in the real world. In this study, only one product (beef mince) and one type of consumer (meat eaters) were considered due to the high cost of conducting large web-based surveys. Therefore, the finding of this study should not be generalised to all food products, nor can they help to understand non-meat eaters’ preferences for heathier food products.

In this study, we focused on assessing consumers’ preferences and WTP for nutrition labels and how they are affected by the presence of other desirable labels such as “Organic” and “Local”. However, not only food attributes and their corresponding labels influence how consumers perceive the usefulness of nutrition labels and health claims. We believe that our knowledge on the use of nutrition labels and health claims would benefit from further research on whether and how factors such as type of diet, social norms, perception of body image and culture affect consumers’ understanding and use of health-related labels and claims. Furthermore, due to the importance of investigating the heterogeneity of consumer preferences and behaviour, we believe that the effect of those factors (e.g., diet, body image and cultural factors) should also be assessed for different consumer segments (e.g., consumers segmented by age group, income level, household composition and purchasing habits).

## Figures and Tables

**Table 1:** Respondents’ sociodemographic characteristics.

Characteristic	UK	Spain
Gender		
Female	60%	60%
Male	40%	40%
Age		
18–24	11%	10%
25–34	16%	10%
35–44	19%	27%
45–54	17%	25%
55+	36%	27%
Employment status		
Employed	61%	44%
Self-employed	8%	13%
Retired	5%	13%
Homemaker	5%	8%
Student	7%	7%
Other	7%	1%
Unemployed	7%	14%
Sub-country (UK)/Region (Spain)		
Scotland	11%	--
England	80%	--
Wales	5%	--
Northern Ireland	3%	--
Northwest (Galicia, Principado de Asturias)	--	8%
Castilla-León	--	5%
North (Cantabria, País Vasco, La Rioja, C. Foral de Navarra)	--	8%
Northeast (Aragón, Islas Baleares, Cataluña)	--	20%
Levante (Comunidad Valenciana, Región de Murcia)	--	14%
Centre-south (Castilla La Mancha, Extremadura, Madrid)	--	21%
Andalucía y Canarias	--	24%

**Table 2:** Attribute levels of beef mince.

Attribute	Levels (UK)	Levels (Spain)
Fat content	**Low**: 3 g per 100 g serving of beef mince	**Low**: 3 g per 100 g serving of beef mince
	**Moderate**: 12 g per 100 g serving of beef mince	**Moderate**: 12 g per 100 g serving of beef mince
	**High**: 21 g per 100 g serving of beef mince	**High**: 21 g per 100 g serving of beef mince
Origin	**Local**: the beef cattle were raised and the beef mince was produced in the UK sub-country (Scotland, England, Wales or Northern Ireland) where the respondent lives	**Local**: the beef cattle were raised and the beef mince was produced in the autonomous region (“Comunidad Autónoma”) where you the respondent lives
	**Rest of the UK/National**: if the beef cattle were raised and the beef mince was produced in the UK but not in the UK sub-country where the respondent lives	**Rest of Spain/National**: if the beef cattle were raised and the beef mince was produced in Spain but not in the autonomous region (“Comunidad Autónoma”) where the respondent lives
	**Imported**: if beef mince was not produced in the UK, but has its origin in an EU (90% of beef imports) or non-EU country (10% of beef imports)	**Imported**: if beef mince was not produced in Spain, but has its origin in an EU (85% of beef imports) or non-EU country (15% of beef imports)
Type of production	**No label**: Beef mince is not labelled as “Organic”	**No label**: Beef mince is not labelled as “Organic”
	**Organic**: if the beef cattle was born and had been raised on organic pasture, had never received antibiotics and growth hormones; was fed only organic feed; and had unrestricted outdoor access	**Organic**: if the beef cattle was born and had been raised on organic pasture, had never received antibiotics and growth hormones; was fed only organic feed; and had unrestricted outdoor access
Greenhouse gas emissions *	**Low**: 5.9 kg of CO_2_e per 500 g of beef mince	**Low**: 5.9 kg of CO_2_e per 500 g of beef mince
	**Moderate**: 19.1 kg of CO_2_e per 500 g of beef	**Moderate**: 19.1 kg of CO_2_e per 500 g of beef
	**High**: 32.2 kg of CO_2_e per 500 g of beef mince	**High**: 32.2 kg of CO_2_e per 500 g of beef mince
Price	£1.50	2.30 €
	£3.00	3.10 €
	£4.50	3.90 €
	£6.00	4.70 €

* Note that this does not include the emissions resulting from the processing and transportation of the meat.

**Table 3:** Estimated marginal utilities and willingness to pay.

Variables	Preferences								Marginal Willingness to Pay				
	Mean (UK)		Std. Deviation (UK)		Mean (Spain)		Std. Deviation (Spain)		Mean (UK)		Mean (Spain)		*p*-Value (Poe Test)
Low Fat	2.700	***	1.606	***	1.819	***	1.252	***	3.41	***	1.94	***	0.00
Moderate Fat	1.769	***	−0.323		1.469	***	0.715	***	2.23	***	1.57	***	0.00
Local	1.222	***	0.784	***	1.512	***	0.973	***	1.54	***	1.61	***	0.31
Rest of the Country/National	1.051	***	0.431	***	1.283	***	0.832	***	1.33	***	1.37	***	0.37
Organic	0.807	***	1.172	***	1.019	***	1.051	***	1.02	***	1.09	***	0.31
Low GHG	1.621	***	−0.410	***	0.901	***	−0.033		2.05	***	0.96	***	0.00
Moderate GHG	0.697	***	0.696	***	1.163	***	0.543	***	0.88	***	1.24	***	0.00
Low Fat and Local	0.197	**	−0.052		0.229	**	1.221	***	0.25	**	0.24	**	0.49
Low Fat and Organic	−0.304	***	−0.728	***	0.270	***	−0.095		−0.38	***	0.29	***	0.00
Low Fat and Low GHG	−0.866	***	0.110		0.499	***	0.012		−1.09	***	0.53	***	0.00
Moderate Fat and Local	−0.067		0.465	*	0.417	***	−0.065		0.00		0.44	***	0.00
Moderate Fat and Organic	0.099		−0.457	**	0.211	**	0.073		0.00		0.22	**	0.28
Moderate Fat and Low GHG	−0.777	***	0.527	***	0.270	**	−0.003		−0.98	***	0.29	**	0.00
Price	−0.456	***	0.828	***	−0.245	***	0.599	***	--		--		--
NoBuyOption	0.372	***	2.282	***	−0.649	***	2.610	***	--		--		--
Number of observations	10,899				10,854				--		--		--
Log likelihood	−11,036.67				−11,671.099				--		--		--
Chi square (χ^2^)	3307.94				3323.69				--		--		--
*p*-value	<0.01				<0.01				--		--		--

Note that (***), (**) and (*) indicate that the corresponding value is statistically significant at (1%), (5%) and (10%) level, respectively. GHG stands for Greenhouse gas emissions. Furthermore, the number of observation is equal to the number of respondents multiplied by the number of choice sets (9 per respondent).

**Table 4:** Heterogeneity of consumers’ WTP–UK.

Claims	Marginal Willingness to Pay										*p*-Value			
	Women		Men		Young		Adults		Elderly		Women vs. Men	Young vs. Adult	Young vs. Elderly	Adult vs. Elderly
Low Fat	3.81	***	3.11	***	3.28	***	3.20	***	4.49	***	0.02	0.43	0.04	0.00
Moderate Fat	2.34	***	2.19	***	2.72	***	1.90	***	3.24	***	0.33	0.06	0.24	0.01
Local	1.75	***	1.40	***	1.42	***	1.35	***	2.56	***	0.05	0.41	0.01	0.01
Rest of the Country/National	1.46	***	1.23	***	1.10	***	1.09	***	2.65	***	0.11	0.49	0.00	0.00
Organic	1.18	***	0.90	***	1.23	***	1.06	***	0.73	***	0.10	0.30	0.12	0.00
Low GHG	2.22	***	1.95	***	2.01	***	2.02	***	2.16	***	0.17	0.50	0.40	0.16
Moderate GHG	1.10	***	0.65	***	1.09	***	0.75	***	1.25	***	0.00	0.06	0.30	0.37
Low Fat and Local	0.33	*	0.16		0.16		0.23		0.32	*	0.25	0.43	0.38	0.01
Low Fat and Organic	−0.52	***	−0.27		0.23		−0.52	***	−0.57		0.17	0.02	0.07	0.41
Low Fat and Low GHG	−1.11	***	−1.13	***	−0.90	*	−1.12	***	−1.16	***	0.47	0.35	0.36	0.45
Moderate Fat and Local	−0.13		−0.05		−0.51		0.02		−0.18		0.39	0.13	0.30	0.46
Moderate Fat and Organic	0.40	**	−0.15		0.46		0.04		0.04		0.02	0.15	0.22	0.33
Moderate Fat and Low GHG	−0.86	***	−1.14	***	−0.91	*	−0.89	***	−1.42	***	0.21	0.48	0.25	0.50

Note that (***), (**) and (*) indicate that the corresponding value is statistically significant at (1%), (5%) and (10%) level, respectively. GHG stands for Greenhouse gas emissions.

**Table 5:** Heterogeneity of consumers’ WTP–Spain.

Claims	Marginal Willingness to Pay										*p*-Value			
	Women		Men		Young		Adults		Elderly		Women vs. Men	Young vs. Adult	Young vs. Elderly	Adult vs. Elderly
Low Fat	1.96	***	1.87	***	2.40	***	1.77	***	2.19	***	0.34	0.04	0.34	0.12
Moderate Fat	1.62	***	1.47	***	1.78	***	1.52	***	1.43	***	0.26	0.26	0.24	0.38
Local	1.62	***	1.58	***	2.07	***	1.52	***	1.48	***	0.41	0.04	0.07	0.44
Rest of the Country/National	1.25	***	1.45	***	1.64	***	1.36	***	0.96	***	0.10	0.14	0.02	0.04
Organic	1.03	***	1.13	***	1.12	***	1.00	***	1.45	***	0.28	0.34	0.17	0.04
Low GHG	0.80	***	1.12	***	1.52	***	0.80	***	1.21	***	0.06	0.02	0.24	0.10
Moderate GHG	1.26	***	1.17	***	1.65	***	1.13	***	1.39	***	0.25	0.01	0.18	0.09
Low Fat and Local	0.11		0.38	**	−0.12		0.28	**	0.28		0.10	0.14	0.19	0.49
Low Fat and Organic	0.24	**	0.30	**	0.27		0.37	***	−0.23		0.37	0.37	0.10	0.02
Low Fat and Low GHG	0.75	***	0.19		0.50		0.56	***	0.42		0.02	0.43	0.45	0.35
Moderate Fat and Local	0.34	**	0.46	***	0.19		0.45	***	0.51		0.31	0.25	0.25	0.43
Moderate Fat and Organic	0.31	**	0.10		0.27		0.30	***	−0.30		0.14	0.46	0.08	0.02
Moderate Fat and Low GHG	0.36	**	0.16		0.22		0.19		0.84	**	0.21	0.47	0.11	0.00

Note that (***) and (**) indicate that the corresponding value is statistically significant at (1%) and (5%) level, respectively. GHG stands for Greenhouse gas emissions.
